# Evaluation of the effect of gentamicin in surgical perfusion solution on cataract postoperative endophthalmitis

**DOI:** 10.1186/s12886-022-02633-2

**Published:** 2022-10-23

**Authors:** Wenjiang Ma, Guanghua Hou, Junfang Wang, Ting Liu, Fang Tian

**Affiliations:** Tianjin Key Laboratory of Retinal Functions and Diseases, Tianjin Branch of National Clinical Research Center for Ocular Disease, Eye Institute, School of Optometry, Tianjin Medical University Eye Hospital, Tianjin, China

**Keywords:** Gentamicin, Endophthalmitis, Cataract, enterococcus

## Abstract

**Objective:**

To evaluate the effect of gentamicin in surgical perfusion solution on endophthalmitis incidence after cataract surgery.

**Methods:**

A retrospective analysis of endophthalmitis incidence was conducted in two groups of patients who underwent cataract surgery, with (Group B) or without gentamicin (Group A) in the surgical perfusion solution. Endophthalmitis incidence, the isolated pathogenic bacteria strains and their antibiotic sensitivity, and the drug-resistant genotype of the pathogens were examined.

**Results:**

The incidence of endophthalmitis in patients of group A was 0.8‰. Thirteen pathogenic bacterial strains were isolated from the patient samples in group A, including 8 strains of *Staphylococcus epidermidis*, 1 *Staphylococcus aureus*, 1 *Streptococcus pneumoniae*, 1 *Streptococcus bovis*, 1 *Enterococcus faecium* and 1 *Morganella sp*. The incidence of endophthalmitis in group B patients was 0.2‰, which was significantly lower than that in group A (P<0.05). Five strains of pathogenic bacteria were successfully isolated, including 2 strains of *Enterococcus faecium*, 1 *Enterococcus faecalis*, 1 *Staphylococcus epidermidis* and 1 *Staphylococcus aureus*. There was no significant difference in the proportion of *Staphylococcus* strains in all isolated bacteria between the two groups (P > 0.05). However, the proportion of *Enterococci* isolated in group B samples was higher than that in group A (P < 0.05). There were more gentamicin-sensitive strains than levofloxacin-sensitive strains identified (P < 0.05). Interestingly, aminoglycoside-inactivating enzyme resistance gene was detected in *Enterococcus* strains.

**Conclusion:**

Our data suggest that gentamicin-containing perfusion solution can reduce the incidence of postoperative endophthalmitis in cataract patients. However, the selective pressure imposed by gentamicin may facilitate the development of aminoglycoside-resistant *Enterococcos* strains.

## Introduction

Endophthalmitis after cataract surgery is a prevalent complication that seriously undermines the visual recovery of the patients [1]. Endophthalmitis is devastating condition resulted from the purulent inflammation of the intraocular fluids, i.e., the vitreous and the aqueous humor. Although the etiology of this condition can be both endogenous and exogenous, it is mostly secondary to intraocular surgeries, injections [2], or penetrating ocular trauma [3]. Acute endophthalmitis appears as a postoperative complication within 1–2 weeks after the surgical intervention, most commonly on the third to fifth postoperative day [4].

Cataract surgery is the most prevalent surgery performed in ophthalmology, but it is also considered as an ocular surface damaging event [5, 6]. A previous study reported that the overall incidence of endophthalmitis after phacoemulsification cataract surgery (PCS) was estimated to be 0.092% [5]. There was also a prevalence of ocular demodicosis and pathogenic conditions on ocular surface after cataract surgery [6]. It is also known that the incidence and severity of dry eye symptoms may increase after cataract surgery. To achieve the best outcome in cataract surgery, a healthy ocular surface is crucial. Patients with frail ocular surface or surface diseases are at higher risk of post-operative complications such as secondary infections [7]. In addition, multiple risk factors have been associated with endophthalmitis after PCS, including the insertion approach of intraocular lens [8], incision location [9], methods of preoperative disinfection of conjunctival sac [10] and patient age [11]. Although the incidence tends to decrease in recent years, the severe outcomes of endophthalmitis after PCS remains as a serious public health threat [5].

*Staphyloccocus epidermidis* is the most common pathogen causing endophthalmitis as a complication after cataract surgery. However, endophthalmitis can be caused also by bacteria, fungi, or viruses [12]. Perioperative cleaning of the conjunctival sac, disinfection of the surgical field with povidone-iodine and the topical use of antibiotics are preventative measures against endophthalmitis after cataract surgery [13, 14]. Antibiotics can be topically applied by instilling antibiotic eye drops, through anterior chamber injection after surgery or by adding in surgical perfusion solution [15, 16]. Intracameral antibiotics administration has been shown to reduce the risk of endophthalmitis [5].

On the other hand, the wide application of antibiotics also causes the development of drug resistance in the pathogens. For example, levofloxacin is a commonly used antibiotic to reduce the risk of endophthalmitis [17]. An increased incidence of levofloxacin-resistant *Staphylococcus* strains has been reported in ophthalmic infection [17], which limits its efficacy in the prevention of endophthalmitis after ocular surgery. In addition, methicillin-resistant *Staphylococcus epidermidis* (MRSE) strains have been found in more than 50% ophthalmic infections [18]. A wide spectrum of antibiotics has been developed for therapeutic use in coping with increased bacterial antibiotic resistance [12]. Recently, more alternatives are available in the market for therapy or the prevention of ocular infections [2, 4, 12].

A previous report suggests that the MIC_90_ (the minimum inhibitory concentration to inhibit 90% of organisms) of gentamicin against *Staphylococcus* is 8 µg/mL [19]. Its bactericidal effect is related to the ratio of peak concentration to the minimum inhibitory concentration (Cmax/MIC). A dose of 80 µg/mL gentamicin in perfusion solution has been adopted in certain surgical procedures, since this practice seems to show a satisfactory sterilization effect [19]. However, there is a lack of data regarding the beneficial effects of gentamicin on the prevention of endophthalmitis in cataract surgery. The aim of this study was to examine endophthalmitis incidence, isolate pathogenic bacteria strains, and characterize antibiotic sensitivity and the drug-resistant genotype in patients who underwent cataract surgery with or without gentamicin in the surgical perfusion solution.

## Materials and methods

### Research subjects

This was a retrospective controlled study. We enrolled all the patients who underwent cataract surgery from Jan 1, 2014 to Dec 31, 2017. Exclusion criteria were: patients without intraoperative intraocular lens implantation; patients with allergy to iodine reagent; patients without complete medical record. All the patients received levofloxacin eye drops 3 times a day for 3 days before surgery. Conjunctival sac was flushed with normal saline on the day of surgery, and 0.5% povidone-iodine was used for disinfection before surgery. After topical anesthesia, phacoemulsification was performed to remove the lens and to implant an intraocular lens. Levofloxacin eye drops were applied three times a day after the operation, which was terminated on day 30.

The enrolled patients were divided into two groups. A total number of 16,408 patients who underwent operation from Jan 2014 to Dec 2015 were assigned as group A (7712 males and 8696 females, average age of 72.8 ± 4.8 years), and no gentamicin was added to the surgical perfusion solution in this group. Endophthalmitis occurred in 13 cases (5 males and 8 females, average age of 74.8 ± 6.3 years) within 30 days after the operation, with a median onset time of 5 days. A total number of 21,469 patients (10,091 males and 11,378 females, average age of 73 ± 3.9 years) who underwent the operation from Jan 2016 to Dec 2017 were assigned as group B, and 80 µg/mL gentamicin was added to the surgical perfusion solution for this group. Endophthalmitis occurred in 5 cases (1 male and 4 females, average age of 72.4 ± 5.9 years.) within 30 days after operation, with a median onset time of 3 days. In case of suspected postoperative endophtalmitis, ophthalmic examination and echo B-scan were performed. The typical symptoms include conjunctival hyperemia and chemosis, corneal edema, tyndall sign and hypopion, exudate deposition on intraocular lens, opacity caused by vitreous infiltration, and severe abscess formation.

### Bacterial culture

Aqueous humor or vitreous humor samples were extracted from patients with endophthalmitis and subjected to pathogenic examination. The specimens were inoculated into the fastidious bacterial enrichment solution immediately after collection. After culturing in a 37 ℃ incubator for 24 h, the samples were then transferred to the blood agar medium for another 48 h at 37 ℃ and Sapaul medium for 7-day culture at 28 ℃. The positive colonies were harvested for microbial identification and drug sensitivity test. If there were no observable colonies identified in the blood agar plate for 48 h or on the Sapaul medium for 7 days, the samples were reported as negative for both bacterial and fungal infections. The above procedures were completed in accordance with strict aseptic techniques to avoid contamination.

### Bacterial identification and drug susceptibility test

VITEK-II automatic bacterial identification/drug susceptibility system (ID-AST card identification) was employed for bacterial identification and drug sensitivity test. Drug susceptibility card (AST-P535, bioMérieux, Marcy l′Etoile, France) was used together with the recommended quality controls: the negative control strain was ATCC29212 (*Enterococcus faecalis*, sensitive to high levels of gentamicin), and the positive control strain was ATCC51299 (*Enterococcus faecalis*, resistant to high levels of gentamicin). The results were interpreted based on the VITEK-II senior expert system identification system standards. As a result, *Enterococci* strains with high-level gentamicin resistance (HLGR) were identified. According to the clinical antibiotic dose in ophthalmic operation, the sensitivity test breakpoint of gentamicin was set as 80 µg/mL and the breakpoint of levofloxacin was set as 4 µg/mL.

### Detection of aminoglycoside inactivation enzyme genes

The aac(6’)-aph(2’’) aminoglycoside inactivating enzyme genes of the identified Enterococcus with HLGR phenotype were examined by PCR method. The DNA sample of the bacteria was extracted using Bacterial Genomic DNA Isolation Kit (Norgen Biotek, Wuhan, China) according to the manufacturer’s instruction. PCR was performed to examine the presence of aminoglycoside inactivation enzyme genes using the following primers: sense primer: 5’-GGTGGTTTTTACAGGAATGCC ATC-3’; antisense primer: 5’-CCCTCTTCATACCAATCCATACC-3 (Shanghai Sangon Bioengineering Co., Ltd, China). The following PCR conditions were used in a 20uL PCR reaction: 94 °C for 5 min, 30 cycles of denaturation at 94 °C for 30s, annealing at 52 °C for 30s, and extension at 72 °C for 60s. The PCR products were analyzed using 1% agarose gel electrophoresis, with a detection target at 642 bp.

### Statistical methods

All analyses were performed by SPSS 22.0 statistical software (SPSS, Inc. Chicago, IL, USA). Data were calculated and compared by X^2^ test, and P < 0.05 was considered to be statistically significant.

## Results

In group A without the application of gentamicin, 13 cases of endophthalmitis (out of 16,395 patients) was recorded and confirmed, with an incidence rate of 0.8‰. In group B with the application of gentamicin, 5 cases of endophthalmitis were confirmed in a total number of 21,469 patients, and the incidence rate was 0.2‰ which was significantly lower than that of group A (p = 0.016).

**Fig. 1:**
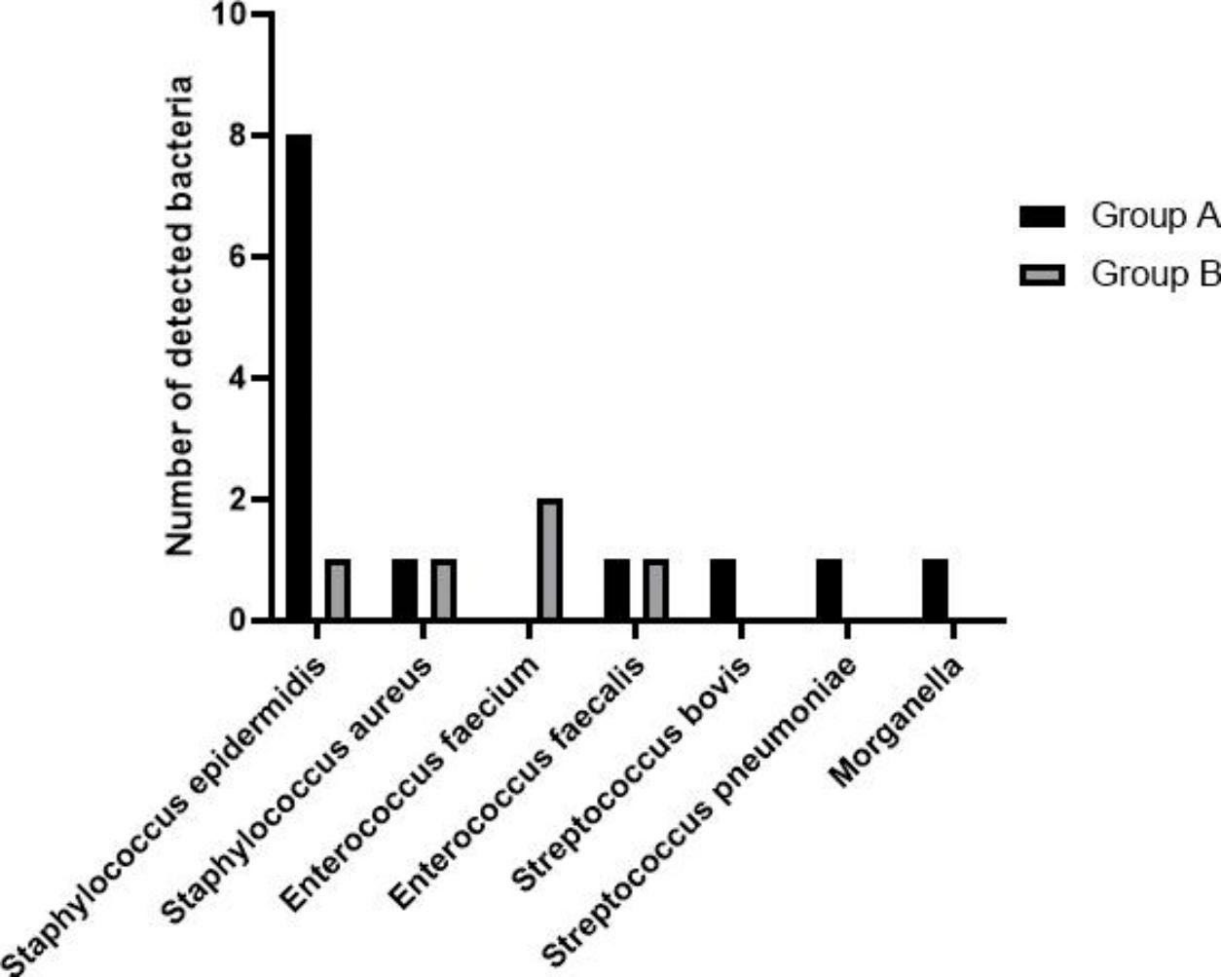
The distribution of pathogenic bacteria identified in the samples of group A and B

Thirteen pathogenic bacterial strains were isolated from the patient samples in group A, including 8 strains of *Staphylococcus epidermidis*, 1 *Staphylococcus aureus*, 1 *Streptococcus pneumoniae*, 1 *Streptococcus bovis*, 1 *Enterococcus faecium* and 1 *Morganella sp*. (Fig. 1). The five strains of pathogenic bacteria in group B samples included 2 strains of *Enterococcus faecium*, 1 *Enterococcus faecalis*, 1 *Staphylococcus epidermidis* and 1 *Staphylococcus aureus* (Fig. 1). In all the samples, there were no detectable fungal infections observed. There was no significant difference in the proportion of *Staphylococcus* among the isolated bacteria strains between the two groups (P = 0.326). However, the proportion of *Enterococcus* strains was significantly higher in the pathogenic bacteria identified in group B (P = 0.044).

**Fig. 2:**
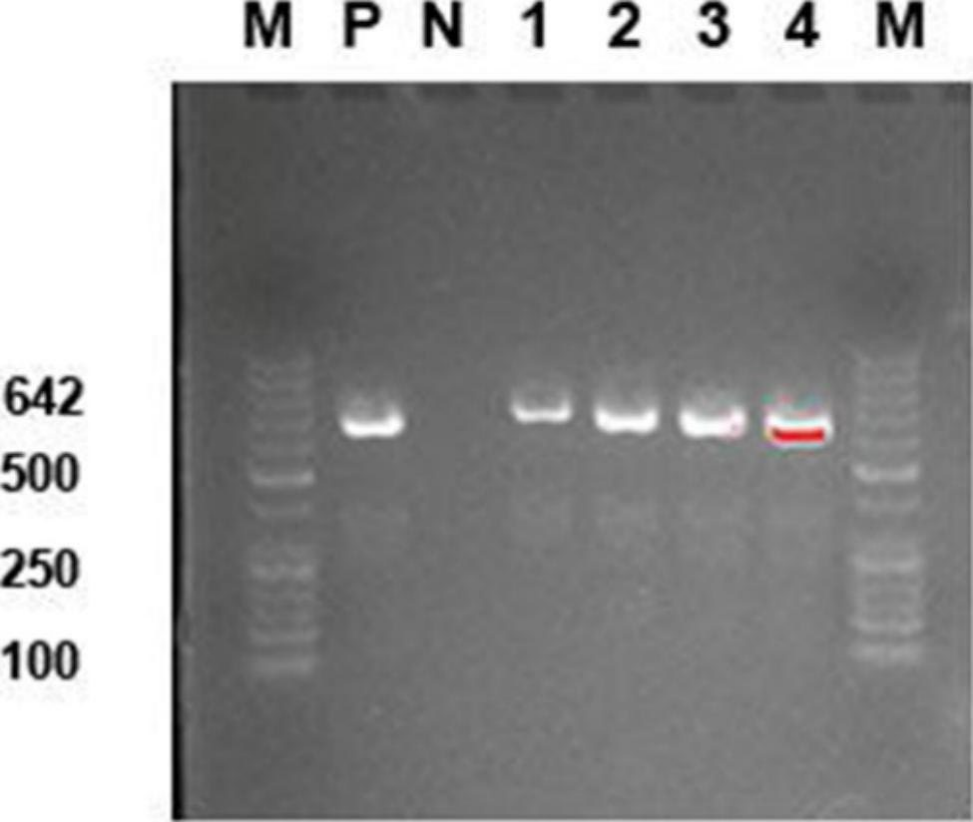
Detection of aac(6’)-aph(2’’) aminoglycoside inactivating enzyme gene by PCR and gel electrophoresis in 4 *Enterococcus* strains. M: Marker; P: positive control_ATCC51299 *Enterococcus faecalis*. N: negative control_ATCC29212 *Enterococcus faecalis*

We also examined the sensitivity of all the isolated strains (18 strains) towards gentamicin and levofloxacin. There were more gentamicin-sensitive strains than levofloxacin-sensitive strains identified (Table 1, P = 0.018). The MICs of levofloxacin ranged from 1 to 32 µg/ml, and the MICs of gentanmicin ranged from 1 to 500 µg/ml (Table 2). Since *Enterococci* with high-level gentamicin resistance (HLGR) phenotype were identified, we further examined whether those strains harbors aminoglycoside-inactivation genes using targeted primers. The ATCC29212 *Enterococcus faecalis* strain (sensitive to high levels of gentamicin) was used as the negative control, and the positive control strain was ATCC51299 (*Enterococcus faecalis*, resistant to high levels of gentamicin). PCR and gel electrophoresis analysis showed that all the four *Enterococcus* strains contained aminoglycoside-inactivation gene (Fig. 2).

**Table 1:** Antibiotic susceptibility analysis of 18 pathogenic strains isolated

Antibiotics	Breakpoint(µg/ml)	Sensitive	Insensitive	Sensitive rate(%)	X^2^	P value
Gentamicin	80	14	4	77.8	7.200	0.018
Levofloxacin	4	6	12	31.6		

**Table 2:** MIC of isolated bacteria to levofloxacin and gentamicin

Group A			Group B		
Levofloxacin Gentamicin			Levofloxacin Gentamicin		
Strains	MIC(µg /mL)	MIC(µg /mL)	Strains	MIC(µg /mL)	MIC(µg /mL)
*Staphylococcus epidermidis 1*	8(R)	8(S)	*Staphylococcus epidermidis*	8(R)	32(S)
*Staphylococcus epidermidis 2*	8(R)	4(S)	*Staphylococcus aureus*	8(R)	32(S)
*Staphylococcus epidermidis 3*	4(S)	32(S)	*Enterococcus faecium 1*	4(S)	500(R)
*Staphylococcus epidermidis 4*	1(S)	1(S)	*Enterococcus faecium 2*	8(R)	500(R)
*Staphylococcus epidermidis 5*	8(R)	2(S)	*Enterococcus faecalis*	16(R)	500(R)
*Staphylococcus epidermidis 6*	16(R)	16(S)			
*Staphylococcus epidermidis 7*	8(R)	4(S)			
*Staphylococcus epidermidis 8*	32(R)	32(S)			
*Staphylococcus aureus*	16(R)	8(S)			
*Enterococcus faecalis*	8(R)	500(R)			
*Streptococcus bovis*	4(S)	4(S)			
*Streptococcus pneumoniae*	4(S)	16(S)			
*Morganella. Sp.*	4(S)	8(S)			

**S: sensitive; R: resistant**

## Discussion

Endophthalmitis after cataract surgery is a serious complication with an incidence ranging from 0.6‰ to 1.4‰. An infection rate greater than 1‰ indicates the insufficiency of perioperative preventive measures [20]. The occurrence of endophthalmitis after cataract surgery is related to the number of bacteria in the intraoperative aqueous humor, the ability of the immune system to restrict pathogenic microorganisms and the effectiveness of perioperative antibiotic intervention [21, 22]. In this retrospective study, without gentamicin being added to the surgical perfusion solution, the postoperative infection rate was 0.8‰ and the confirmed incidence of endophthalmitis was close to the level reported in the literature. When gentamicin was present in the perfusion solution, the incidence of postoperative endophthalmitis was reduced to 0.2‰. These data suggest a beneficial effect of gentamicin in the prevention of postoperative endophthalmitis.

Previous studies have demonstrated that preoperative instillation of antibiotic solution, conjunctival sac irrigation and preoperative povidone-iodine disinfection of the conjunctival sac can significantly reduce the bacterial load in the surgical field [23]. However, it is impossible to maintain a sterile state in the surgical field during the whole procedures of surgery [24]. Bacteria colonized in the margin of eyelid, meibomian glands and conjunctival sac can spread to the surgical field or enter the anterior chamber through surgical instruments [25–27]. Perioperative use of antibiotics reduces the incidence of endophthalmitis after cataract surgery [28]. The type of antibiotic, the mode of use and the pharmacokinetic parameters of topical antibiotics can all affect the anti-bacterial outcome and bacterial spectrum in endophthalmitis after surgery [29, 30]. We also observed that the spectrum of pathogenic bacteria in endophthalmitis samples changed when gentamicin was present in the perfusion solution. Particularly, the proportion of *Enterococcus* strains showed a significant increase.

Perioperative instillation of fluoroquinolone is a common approach for preventing endophthalmitis after cataract surgery [31]. The concentration of different quinolone antibiotics in the anterior chamber can reach to 1–4 µg/ml [32–35]. To reduce endophthalmitis incidence, intracameral injection of cefuroxime, moxifloxacin and vancomycin in the surgical perfusion solution has been adopted in clinical practice. However, adverse reactions due to use of antibiotics are also a serious concern [36–39]. Gentamicin is a concentration-dependent antibiotic with superior bactericidal effect on *Staphylococcus*. Here, we reported that adding gentamicin to the perfusion solution could reduce cataract postoperative endophthalmitis incidence, and no obvious side effects were observed. When gentamicin is administered systemically, significant side effects can occur if Cmax exceeds 12 µg/mL [40, 41]. The safe concentration of gentamicin in the perfusion solution was much higher than the MIC_90_, suggesting that an excellent bactericidal effect on coagulase-negative pathogens such as *Staphylococci* strains. In this study, the drug sensitivity breakpoints of levofloxacin and gentamicin were 4 µg/mL and 80 µg/ml, respectively. Most pathogens (except 4 strains of *Enterococci*) were sensitive to 80 µg/mL gentamicin. One strain of MRSE and one strain of MRSA were detected in group B samples, however their MICs of gentamicin were lower than 80 µg/mL. This suggests that postoperative endophthalmitis can be caused by factors other than antibiotic precaution, such as the immune condition or intraocular pharmacokinetics of antibiotics.

The incidence of enterococcal endophthalmitis after cataract surgery tends to increase recently. *Enterococci* are more resistant to the fourth-generation fluoroquinolones than coagulase-negative *Staphylococci* [26, 42]. Aminoglycoside inactivating enzyme encoded by the aac(6’)-aph(2’’) gene can confer resistance to high concentrations of gentamicin [43]. In our study, we identified four strains of *Enterococci* which were highly resistant to high concentrations of gentamicin. Interestingly, the aac(6’)-aph(2’’) gene was detected in all four gentamicin-resistant strains. Thus, the application of gentamicin may serve as a selective pressure to facilitate the domination of strains harboring gentamicin-resistant genes.

Our studies have been limited by the numbers of pathogenic bacteria isolated in both groups. We reasoned that the use of different nutrient-rich culture methods may increase the recovery of fastidious pathogenic bacterial strains. In addition, the potential gentamicin-inactivating genes should be cloned and transferred to a sensitive strain to confirm its biological function.

In conclusion, the types and application mode of antibiotics in the perioperative period of cataract surgery affect the incidence of postoperative endophthalmitis and the spectrum of pathogenic bacteria. We reported that gentamicin at a safe concentration can reduce the incidence of endophthalmitis after cataract surgery. However, the presence of gentamicin may also serve as selective pressure to provide survival advantages of gentamicin-resistant *Enterococci*. Therefore, a combination of antibiotics with different modes of actions may minimize the chance of drug resistance, which warrants future clinical validation.

## Data Availability

All data included in this study are available upon request by contact with the corresponding author.
